# Exposure to azithromycin and the effect of co-administration of rifampicin in patients with non-tuberculous mycobacterial disease

**DOI:** 10.1093/jac/dkag206

**Published:** 2026-06-13

**Authors:** M P Rodgers, R Stemkens, V N Dahl, A van Laarhoven, W Hoefsloot, A Lemson, J van Ingen, R E Aarnoutse

**Affiliations:** Department of Pharmacy, Pharmacology & Toxicology, Radboudumc Community for Infectious Diseases, Radboud University Medical Center, Nijmegen, The Netherlands; Radboudumc, Department of Medical Microbiology, Radboudumc Community for Infectious Diseases, Radboud University Medical Center, Nijmegen, The Netherlands; Department of Pharmacy, Pharmacology & Toxicology, Radboudumc Community for Infectious Diseases, Radboud University Medical Center, Nijmegen, The Netherlands; Department of Infectious Diseases, Aarhus University Hospital, Aarhus, Denmark; Radboudumc, Department of Internal Medicine, Radboudumc Community for Infectious Diseases, Radboud University Medical Center, Nijmegen, The Netherlands; Department of Pulmonary Diseases, Radboudumc Community for Infectious Diseases, Radboud University Medical Center, Nijmegen, The Netherlands; Department of Pulmonary Diseases, Radboudumc Community for Infectious Diseases, Radboud University Medical Center, Nijmegen, The Netherlands; Radboudumc, Department of Medical Microbiology, Radboudumc Community for Infectious Diseases, Radboud University Medical Center, Nijmegen, The Netherlands; Department of Pharmacy, Pharmacology & Toxicology, Radboudumc Community for Infectious Diseases, Radboud University Medical Center, Nijmegen, The Netherlands

## Abstract

**Objectives:**

Azithromycin is a key drug in the treatment of most non-tuberculous mycobacterial (NTM) diseases. Its exposure may be decreased by rifampicin co-administration, but to what extent is largely unknown. We measured azithromycin exposure in an NTM disease patient population and quantified the effect of rifampicin co-administration.

**Methods:**

We retrospectively collected plasma azithromycin area-under-the-curve from 0 to 6 hours after administration in mg/L*hours (AUC_0-6h_), peak (C_max_), and trough (C_min_) concentrations from the TDM service at Radboudumc, The Netherlands. Azithromycin exposure measures were compared between patients with and without concurrent rifampicin use, and within patients who had rifampicin stopped during treatment.

**Results:**

We analysed data of 130 patients, of whom 59% had NTM pulmonary disease. The azithromycin geometric mean of AUC_0-6h_ in patients with (*n* = 48) and without (*n* = 82) rifampicin were 0.90 versus 1.83 mg/L*h, C_max_ 0.22 versus 0.46 mg/L, and C_min_ 0.043 versus 0.13 mg/L, respectively. A within-patient comparison in 14 subjects showed geometric means of AUC_0-6h_, C_max_, and C_min_ (90%-CI) with rifampicin were 62% (45%–74%), 58% (38%–72%) and 66% (48%–77%) lower than without rifampicin. Interventions based on TDM enabled a strong increase in exposure to azithromycin. No association between azithromycin exposure and disease outcomes was shown, but the number of patients in these analyses was small.

**Conclusions:**

This study provides new population exposure data for TDM of azithromycin in NTM disease. Rifampicin co-administration reduces azithromycin exposure by at least half, underscoring the need for upfront azithromycin dose adjustment, application of TDM, or considering alternative drugs for rifampicin, also considering controversy around its effectiveness and adverse effects.

## Introduction

Non-tuberculous mycobacterial (NTM) disease is increasingly recognized as an important opportunistic infection of humans. Its most frequent manifestation is NTM pulmonary disease (NTM-PD),^1^ but extrapulmonary manifestations include lymphadenitis, skin and soft tissue infections, and disseminated disease in the severely immunocompromised. NTM disease is notoriously difficult to treat and shows high rates of treatment failure and adverse effects.^2,3^ Azithromycin is often the cornerstone of NTM treatment, and high azithromycin exposures have been associated with improved microbiological outcomes.^4^ The effectiveness of azithromycin may be compromised by co-administration of rifampicin, which is known to decrease azithromycin exposure.^4,5^ Still, the pharmacokinetic interaction between these two drugs is poorly quantified.

Therapeutic drug monitoring (TDM), i.e. individualized drug dosing based on measurement of plasma or serum drug concentrations, has emerged as a valuable tool for optimising antibiotic therapy in NTM disease. Despite the critical role of azithromycin, no clinically validated plasma concentration targets for this drug exist. Current dosing strategies rely on population (average) exposure measures as targets. However, population average exposure data are rarely reported and have so far been based on very limited sampling that does not allow for an accurate estimation of total exposure (AUC), peak (C_max_) or trough (C_min_) concentrations.^4,5^

This study aimed to address these gaps by investigating azithromycin exposure in a population of NTM patients. Specifically, we sought to: (1) describe population-level exposure measures for azithromycin to inform clinical dosing targets; (2) quantify the effect of rifampicin co-administration on azithromycin pharmacokinetics; and (3) identify other potential predictors of azithromycin exposure, such as age, sex, and dose per kilogram. Additionally, we conducted an explorative evaluation to determine whether exposure measures and dose adjustments due to TDM were associated with microbiological and clinical outcomes.

## Patients and methods

### Data gathering

This retrospective study included adult patients who received azithromycin daily at a known dose and for whom azithromycin TDM was performed as part of NTM disease management at Radboudumc, Nijmegen, The Netherlands, between 2021 and January 2025. We additionally included patients from other centres whose samples were sent to Radboudumc for TDM. TDM is performed for all patients at Radboudumc with NTM-PD, and on indication for patients with other NTM-disease manifestations. Patients were excluded if rifampicin use was unknown, TDM was performed before steady state for azithromycin was achieved, or if implausible concentrations suggested errors in the TDM process.

Data were collected on patient demographics (age, sex, and weight), NTM species identification, clinical manifestation (i.e. pulmonary, extrapulmonary, or disseminated), azithromycin dose, rifampicin co-administration, azithromycin pharmacokinetic measures (AUC_0-6h_, C_max_, and C_min_, microbiological outcome (‘cure’ defined as negative follow-up cultures)^6^ and clinical outcome (classified according to the descriptions in clinical notes by the treating physician). Furthermore, data were collected on whether azithromycin TDM led to changes in azithromycin dosing or cessation of rifampicin.

Only the first TDM assessment of azithromycin treatment at steady state (i.e. after at least two weeks of treatment, which is roughly 4–5 times the half-life of 2–4 days)^7^ was included in the analysis for each patient. A subset of patients was identified in whom azithromycin exposure was assessed both with and without concurrent administration of rifampicin. In these cases, azithromycin concentrations without rifampicin co-administration were measured at least two weeks after rifampicin discontinuation to assess changes in exposure to azithromycin, without a change in the dose of azithromycin.

The study was assessed by the ethical review board of Radboud University Medical Center (Radboudumc) as not subject to the Medical Research Involving Human Subjects Act (WMO) (Filenumber: 2025-18037).

### Therapeutic drug monitoring (TDM) setting at Radboudumc

In the TDM service at Radboudumc, blood samples are collected before the next azithromycin dose (T_0_), and at 2, 4, and 6 hours (T_2_, T_4_, and T_6_, respectively) after administration. Patients from other centres in the Netherlands were sampled in the same way. Total (protein-bound plus unbound) plasma concentrations of azithromycin are determined using an LC-MS/MS assay, which is validated according to European Medicines Agency and USA Food and Drug Administration guidelines. The accuracy of the assay is between 98% and 101%, depending on the concentration level. The intra- and inter-assay coefficients of variation vary from 1.9% to 3.9% and from 0% to 2.9%, respectively, over the method's range of 0.015 mg/L to 3.0 mg/L.

Upon measurement of individual azithromycin plasma concentrations, the AUC_0-6h_ is calculated using the linear trapezoidal rule. C_max_ and C_min_ (at T_0_) are assessed by visual inspection. The individual azithromycin AUC_0-6h_, C_max_ and C_min_ values are compared to previously described population values,^5^ and dosing advice is provided to the clinician.

### Data analyses

Statistical analyses were performed using IBM SPSS Statistics 29 (SPSS Inc.) AUC_0-6h_, C_max_, and C_min_ values were log-transformed for all tests.

Geometric means and ranges were calculated for azithromycin AUC_0-6h_, C_max_, and C_min_, stratified by rifampicin co-administration status.

The effect of rifampicin on azithromycin exposure was assessed using a between-group t-test on log-transformed measures in patients with and without rifampicin co-administration. Ratios of geometric means and corresponding 95% CI were calculated to quantify the differences. The proportion of patients in whom C_max_ ≥ 0.4 mg/L was achieved was assessed, as this may be a target that is associated with treatment effectiveness.^4^

A within-subject comparison, using a bioequivalence approach (recommended for drug interactions),^8^ was performed on the subset of patients who had azithromycin concentrations measured with and without rifampicin. This entailed calculating the geometric means of ratios per patient for each exposure measure (AUC_0-6h_, C_max_, and C_min_). If the 90% CI for the geometric mean ratio was entirely outside 80%–125% limits, the results indicated inequivalence, that is, a pharmacokinetic drug interaction.

Univariate linear regression analyses were performed in the with- and without-rifampicin groups to determine whether dose per kg reliably predicts azithromycin exposure, using pharmacokinetic measures as dependent variables. Next, multiple linear regression analyses were performed to explore predictors of azithromycin exposure. Predictors (independent variables) were age, sex, rifampicin co-administration, and azithromycin dose per kg, entered using a stepwise approach. A *P* value of <0.05 was considered statistically significant.

Subsequently, we assessed how many patients' therapy with azithromycin or rifampicin was changed based on TDM results and whether these changes were effective from a pharmacokinetic point of view.

Finally, differences in exposure measures between patients based on microbiological outcome (categorized as ‘cure’ or ‘failure’, based on NTM-NET outcome definitions^6^) and based on clinical outcome (categorized as ‘improvement’ or ‘stable/worsening‘) were explored with independent samples t-tests. Chi-squared tests were used to assess whether a C_max_ ≥ 0.4 and/or an increase in azithromycin exposure (either by increasing azithromycin dose and/or discontinuing rifampicin) were associated with microbiological and clinical outcomes. These tests involving outcomes were performed in two subgroups: (1) patients in whom therapy remained unchanged after TDM (if therapy was changed, the exposures measured before therapy change would not be relevant anymore) and (2) the subgroup with NTM-PD (to create a more homogeneous group).

## Results

### Patient characteristics

In total, 130 patients were included for analysis, after exclusion of 13 individuals: six due to unknown rifampicin use, two due to thrice weekly azithromycin dosing, two due to implausible azithromycin concentrations suggestive of errors in the TDM process, one due to unknown azithromycin dose, one due to TDM being performed before steady state, and one due to being a child. The patients’ baseline characteristics are presented in Table 1.

**Table 1.** Characteristics of patients included in the cohort stratified by rifampicin co-administration

	Without rifampicin (*n* = 82)	With rifampicin (*n* = 48)
Male, *n* (%)	34 (41)	28 (58)
Age in years, median, mean (interquartile range)	64 (53–71)	60 (49–69)
Weight in kg, median (interquartile range)	64.2 (55.5–80.1)	63.4 (54.6–71.0)
NTM species, *n* (%)		
* M. avium* complex	43 (52)^a^	30 (63)^b^
* M. abscessus*	18 (22)	—
* M. chelonae*	8 (9.8)	—
* M. malmoense*	1 (1.2)	4 (8.3)
* M. genavense*	1 (1.2)	3 (6.3)
Mixed infection	3 (3.7)^c^	2 (4.2)^d^
Other	8 (9.8)^e^	9 (19)^f^
Disease manifestation, *n* (%)		
Fibrocavitary PD	26 (32)	23 (48)
Nodular-bronchiectatic PD	15 (18)	7 (15)
Other NTM-PD	5 (6)	1 (2.1)
Disseminated	11 (13)	9 (19)
Skin and soft tissue infections	11 (13)	3 (6.3)
Other	14 (17)^g^	5 (10)^h^
Microbiological outcome, *n* (%)^i^		
Cure	49 (60)	33 (69)
Failure	21 (26)	11 (23)
Unknown/no follow-up	12 (15)	4 (8.3)
Clinical outcome, *n* (%)^j^		
Improvement	54 (66)	32 (67)
Stable	2 (2.4)	2 (4.2)
Worsening	22 (27)	11 (23)
Unknown/no follow-up	4 (4.9)	3 (6.3)

SD, standard deviation; PD, Pulmonary disease; NTM-PD, non-tuberculous mycobacterial pulmonary disease not otherwise specified.

^a^25 *M. avium*, 10 *M. chimaera*, 5 *M*. *intracellulare*, 1 *M. colombiense*, 1 *M. paraintracellulare*, 1 *M. yongonense.*

^b^13 *M. avium*, 10 *M. chimaera*, 3 *M. intracellulare*, 2 *M. yongonense*, 1 *M. colombiense,* 1 *M. avium and M. chimaera combined*.

^c^2 *M. intracellulare* and *M. abscessus,* 1 *M. intracellulare* and *M. chimaera.*

^d^2 *M. avium* and *M. kansasii.*

^e^1 *M. celatum*, 1 *M. haemophilum*, 1 *M. mantenii*, 1 *M. marinum*, 1 *M. paragordonae*, 1 *M. simiae*, 1 *M. szulgai*, 1 *M. tilburgii*.

^f^2 *M. shimoidei*, 1 *M. haemophilum*, 1 *M. heraklionense*, 1 *M. kansasii*, 1 *M. kumamotonense*, 1 *M. kyorinense*, 1 *M. tilburgii*, 1 *M. xenopi*.

^g^1 arthritis, 1 breast prosthesis, 2 cervical lymphadenitis, 1 otitis, 1 otomastoiditis and skull osteomyelitis, 1 peritoneal dialysis insertion site infection, 1 peritonitis associated with dialysis, 1 peritonitis associated with catheter infection, 1 septic arthritis and osteomyelitis, 1 skin and osteomyelitis, 1 skin, bone and lymph node infection, 1 splenic abscess, 1 unknown.

^h^1 arthritis, 1 intestinal infection, 1 meningo-encephalitis, 1 spondylodiscitis, 1 tenosynovitis, arthritis, possible osteomyelitis and abscess of hand.

^i^Defined as negative follow-up culture(s) or negative follow-up polymerase chain reaction (2 cases) at the end of treatment.

^J^was determined at the end of treatment.

### Exposure to azithromycin and drug interaction with rifampicin

Overall, the mean daily dose of azithromycin was 7.2 mg/kg. Patients on rifampicin received a higher mean dose of azithromycin of 7.9 mg/kg as opposed to those without (6.8 mg/kg), possibly reflecting higher dosing due to anticipation of the interaction. Despite receiving a higher dose, geometric mean azithromycin exposures were more than 50% lower in the group receiving rifampicin (see Table 2).

**Table 2.** Azithromycin exposure measures and drug interaction with rifampicin (between-patient comparison)

	Without rifampicin (*n* = 82)^a^	With rifampicin (*n* = 48)^a^	Geometric Mean Ratio (95% CI) with/without rifampicin
Daily azithromycin dose in mg/kg	6.8 (2.3–11.9)	7.9 (3.5–17.9)	1.16 (1.04–1.29)
AUC_0-6h_ in h*mg/L	1.83 (0.18–8.36)	0.90 (0.08–2.96)	0.49 (0.37–0.64)
C_max_ in mg/L	0.46 (0.04–2.35)	0.22 (0.02–1.01)	0.48 (0.36–0.64)
C_min_ in mg/L	0.13 (0.01–0.56)	0.043 (0.00–0.52)	0.33 (0.23–0.46)
C_max_ ≥ 0.4 mg/L %	68	25	

^a^Geometric mean values and minimum-maximum values, apart from dose (mean values) and C_max_ ≥ 0.4 mg/L (percentage).

**Figure 1.**
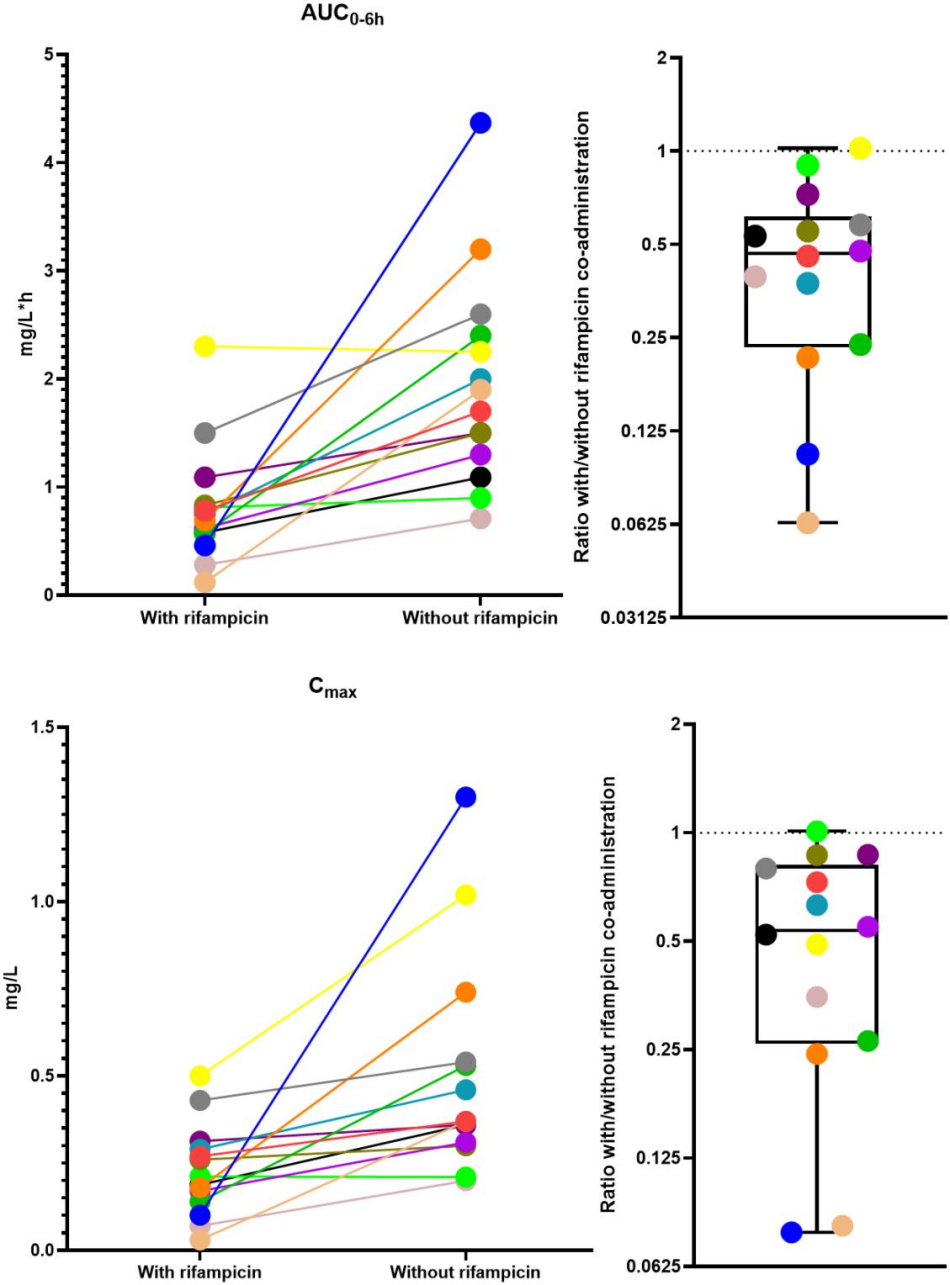

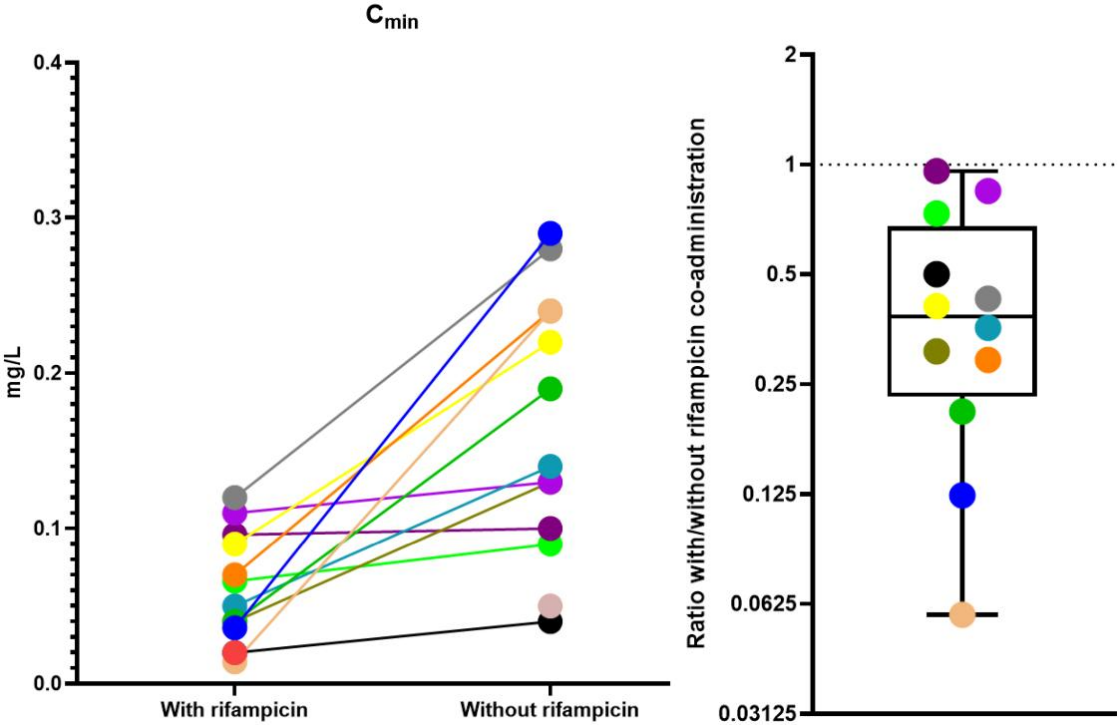
Individual changes in steady-state azithromycin exposure measures with and without co-administration of rifampicin. Each set of two dots connected by a line (and each colour) represents an individual patient. Figures on the left show absolute values, while boxplots on the right show within-patient exposure ratios on a log 2-scale. Solid horizontal lines represent quartiles, and the dashed line represents a ratio of 1 (no difference).

A within-patient comparison could be performed in 14 patients, whose azithromycin concentration measurements were performed with and without concurrent rifampicin use. In 11 patients, azithromycin concentration was measured first with and subsequently without rifampicin. In three patients, this was the opposite. Again, the azithromycin exposures were more than 50% lower with concomitant rifampicin. The 90% CI of the geometric mean ratios were outside the bioequivalence range of 80–125% (Table 3). Intra-individual changes in exposure to azithromycin varied substantially between patients, ranging from practically no change to a 94% lower exposure to azithromycin when combined with rifampicin (Figure 1).

**Table 3.** Azithromycin exposure measures and drug interaction with rifampicin (within-patient comparison, *n* = 14)

	Without rifampicin^a^	With rifampicin^a^	Geometric Mean Ratio (90% CI) with/without rifampicin
AUC_0-6h_ in h*mg/L (*n* = 14)	1.75 (0.71–4.37)	0.66 (0.12–2.30)	0.38 (0.26–0.55)
C_max_ in mg/L (*n* = 14)	0.44 (0.20–1.30)	0.18 (0.03–0.50)	0.42 (0.28–0.62)
C_min_ in mg/L (*n* = 12)	0.14 (0.04–0.29)	0.049 (0.01–0.12)	0.34 (0.23–0.52)

^a^Geometric mean values and minimum-maximum values.

### Predictors of exposure to azithromycin

Dose per kg alone was a significant, but not a good, predictor of azithromycin exposure in patients with or without rifampicin. Univariate regression analysis showed a weak relationship between dose per kg and AUC_0-6h_, C_max,_ and C_min_, with R^2^ (determination coefficient, explained variance) all <0.4. See Table S1 (available as Supplementary data at *JAC* Online) and Figure S1.

In multivariate regression, rifampicin co-administration and azithromycin dose per kg were both significant predictors of AUC_0–6h_, C_max_, and C_min_ (*P* < 0.001), while age and sex were not. The determination coefficient of the regression models ranged from 0.36 to 0.39, and rifampicin co-administration had the most influence in predicting exposure to azithromycin (see Table S2). After adjusting for azithromycin dose, rifampicin co-administration was associated with a 59%, 60%, and 74% reduction in AUC_0–6h_, C_max_, and C_min_, respectively.

### Therapy changes caused by TDM

A change in either azithromycin or rifampicin dosing as a direct result of azithromycin TDM was made in 54 out of 130 (42%) of patients. Azithromycin dosing was adjusted in 44 out of 130 (34%) patients. Dosing was increased in 24 (18%) of these patients and decreased in 17 (13%). Regarding the remaining three patients, therapy was switched to clarithromycin in two patients, and in one patient, the mode of administration was changed (from crushed tablets, administered via a feeding tube, to oral suspension). Rifampicin was stopped as a direct result of azithromycin TDM in 17 out of 48 (35%) patients treated with rifampicin. This typically occurred when azithromycin levels were low, and clofazimine was often started instead. In 7 (5.4%) patients, both azithromycin dosing was changed, and rifampicin was stopped as a result of TDM.

TDM data were available for 15 patients after increasing azithromycin and/or stopping rifampicin. The intervention increased the geometric means of AUC_0-6h_, C_max_, and C_min_ from 0.58 h*mg/L, 0.15 mg/L, and 0.043 mg/L to 1.55 h*mg/L, 0.37 mg/L, and 0.12 mg/L, respectively.

### Microbiological and clinical outcomes related to TDM

Clinical and microbiological outcomes are presented in Table 1. No relationship was found between azithromycin exposure levels and microbiological outcomes (*n* = 63, 48 patients with ‘cure’ and 15 patients with ‘failure’) or clinical outcomes (*n* = 64, 48 patients with ‘improvement’ and 16 patients with ‘stable/worsening’) in patients in whom therapy remained unchanged after TDM, nor in the subgroup with MAC-PD (in total *n* = 26 and *n* = 27 patients). Similarly, no relationship was found between achieving C_max_ ≥ 0.4 mg/L and outcomes in these two groups. There was a positive but not statistically significant trend towards better microbiological and clinical outcomes in patients where azithromycin dosing was increased and/or rifampicin halted due to TDM (odds ratio and 95% CI 1.32 [0.52–3.33], *n* = 114, and 1.86 [0.72–4.78], *n* = 120, for good microbiological and clinical outcomes, respectively). The same was found in the subgroup with MAC-PD (odds ratio and 95% CI 1.91 [0.52–7.09], *n* = 54, and 1.83 [0.50–6.78], *n* = 56). Considering the number of patients in each subgroup, all these concentration-response analyses were probably underpowered.

## Discussion

This study provides population (average) azithromycin exposure data for patients with NTM disease, leading to TDM targets of 1.8 h*mg/L for AUC_0-6h_, 0.46 mg/L for C_max_, and 0.13 mg/L for C_min_. We found considerable variation in azithromycin exposure between patients (Tables 2 and 3, Figure 1), showing that exposure levels cannot accurately be predicted based on dosage alone. On the basis of between-patient as well as within-patient observations, we established that the interaction between rifampicin and azithromycin is much stronger than previously described, resulting in 50–60% lower exposures to azithromycin in patients treated with rifampicin. Interventions based on TDM enabled a strong increase in exposure to azithromycin.

Current guidelines recommend azithromycin over clarithromycin for NTM disease, in part based on previous data showing less interaction with rifampicin.^5,9^ The most extensive pharmacokinetic data on azithromycin currently available describe a cohort of 481 patients with MAC-PD from Denver, USA.^5^ The mean azithromycin dose administered was 6.7 mg/kg, similar to the 7.2 mg/kg in our cohort. Exposure levels were similar for the populations without rifampicin in Denver and the Netherlands (mean AUC_0-6/7h_ of 1.7 mg/L*h versus geometric mean AUC_0-6h_ of 1.8 mg/L*h and mean C_max_ of 0.35 mg/L versus geometric mean of 0.46 mg/L, respectively). However, a large difference was seen in the rifampicin group, with much lower azithromycin exposures in the current study. We observed that, after adjustment for higher azithromycin mg/kg dosing, rifampicin was associated with a reduction of 59%, 60%, and 74%. In contrast, the Denver study reported only a 24% decrease in azithromycin AUC_0-6/7h_ and a 23% decrease in C_max_. Several factors might contribute to this difference. First, the Denver results were based on two sampling time points, while our study used four, improving the accuracy of AUC_0-6h_ and C_max_ estimations. Second, the study in Denver used arithmetic means as a measure of central tendency for their exposure measures, whereas we used geometric means, as pharmacokinetic data are typically not normally distributed. Finally, the cohort in Denver mostly included women with nodular-bronchiectatic pulmonary disease, a subgroup that was less represented in our cohort. Although this indicates a difference in patient populations, it is unclear why this would affect exposure levels. Rifampicin dosing was not recorded in our study, but the standard dose is approximately 10 mg/kg (following weight-banded dosing; 450 mg if <50 kg and 600 mg if >50 kg), similar to the 9.62 mg/kg in the Denver cohort, making rifampicin dosing an unlikely explanation for the observed difference.

Another publication from South Korea has described azithromycin pharmacokinetics in a cohort of 132 patients with NTM disease and quantified the extent of this drug-drug interaction, albeit based on C_max_ only. This study observed a 58% lower median C_max_ in patients receiving concurrent rifampicin, a result more in line with our findings.^4^

Importantly, we were able to evaluate the effect of rifampicin co-administration on azithromycin within the same individuals, offering the highest-quality evidence for quantifying this drug-drug interaction. Interestingly, the extent of interaction varied widely between patients. Little is known about the mechanism of interaction between rifampicin and azithromycin. For clarithromycin, the interaction is postulated to be the result of CYP3A4 induction by rifampicin.^10^ This mechanism is unlikely for azithromycin, as it is unaffected by other CYP3A4 inducers, such as carbamazepine, or inhibitors such as indinavir.^11^ Rifampicin is also an inducer of CYP-enzymes 1A2, 2B6, 2C8, 2C9 and 2C19, but azithromycin is not known to be a substrate for these enzymes.^12^ However, azithromycin has been described as a substrate of P-glycoprotein (PgP),^11,13^ and rifampicin as an inducer of this enzyme.^11,14^ Induction of PgP may therefore explain or contribute to this interaction.

We showed that azithromycin exposure is unpredictable at the individual level and that performing TDM frequently impacts clinical decision-making, as evidenced by therapy adjustments in 42% of cases. We did not find a significant association between increasing azithromycin exposure after TDM and outcomes, although a positive trend was observed. This supports the need for more definitive research on this question and with much larger patient numbers, as our concentration-response analyses were likely to be underpowered. In this way, real target (rather than reference) values for exposure measures may be established, as current data is limited. In the South Korean cohort, a C_max_ of ≥0.4 mg/L was associated with favourable microbiological outcomes in MAC-PD, though only in the subgroup on daily treatment.^4^ In our study, two-thirds of patients without rifampicin and only one-quarter with rifampicin reached this threshold. The validity of targeting a C_max_ of ≥0.4 mg/L based on one study is controversial, and we were unable to replicate this finding. Hollow fibre model studies have suggested an AUC/MIC ratio of 7.51 for success, attainable only by 8 gram per day doses of azithromycin.^15^ Current exposures may thus, even with TDM, be at the very bottom of the dose-response curve. However, regardless of the exact optimal target exposures, our findings provide robust within-patient pharmacokinetic evidence that rifampicin substantially reduces azithromycin exposure. This suggests that rifampicin co-administration may negatively impact the prospect of achieving favourable outcomes. Consequently, azithromycin dose escalation or replacement of rifampicin may be explored as a means to improve outcomes.^16^ A possible alternative to rifampicin is clofazimine, which exhibits fewer drug-drug interactions and has shown comparable results to rifampicin for MAC-PD.^17^

A limitation of this study is its retrospective nature. Although azithromycin TDM is the standard of care for NTM-PD at Radboudumc, the inclusion of patients with extrapulmonary disease, where TDM is only performed on indication, may have led to selection bias. Additionally, patients are sometimes transferred to our centre due to a high disease and treatment complexity, which may reflect underlying issues such as drug failure or toxicity, potentially reflecting abnormal exposure levels. Secondly, we evaluated a total exposure up to 6 hours post-dose (AUC_0-6h_), as this was our reference,^5^ rather than a more comprehensive AUC_0-24h_, which would be preferable for evaluation of both TDM and drug-drug interactions. Thirdly, our number of participants was probably too low to assess relationships between exposures and response. Fourthly, no standardized criteria for clinical outcomes exist, outcomes were retrieved from physician notes in the electronic medical records. Lastly, the ethnicity of patients was not recorded.

In conclusion, this study provides higher-quality population (average) azithromycin exposure data for patients with NTM disease. Until clinically validated thresholds are established, population-level exposures remain the best targets for TDM. The study provides new and robust evidence of a clinically significant interaction between azithromycin and rifampicin. Rifampicin reduced azithromycin exposure by at least 50–60%, reinforcing the need for upfront azithromycin dose adjustment, or, given controversy around its effectiveness and adverse effects, consideration of alternative drugs for rifampicin.

## Supplementary Material

dkag206_Supplementary_Data

## Supplementary data

Figure S1 and Tables S1 and S2 are available as Supplementary data at *JAC* Online.
