# Supplementary material for: Exposure to azithromycin and the effect of co-administration of rifampicin in patients with non-tuberculous mycobacterial disease
Source: J Antimicrob Chemother. 2026 Jun 13;81(7):dkag206. doi: 10.1093/jac/dkag206 (PMC13263526; doi:10.1093/jac/dkag206)
Supplement: dkag206_Supplementary_Data [file dkag206_supplementary_data.docx]

Supplementary tables and figures

# Table S1. Univariate linear regression for prediction of azithromycin exposure using log dose per kg.

|  | R-squared | | Standard error of the estimate | | p-value | |
| --- | --- | --- | --- | --- | --- | --- |
|  | Without rifampicin | With rifampicin | Without rifampicin | With rifampicin | Without rifampicin | With rifampicin |
| logAUC_0-6h_ | 0.365 | 0.117 | 0.253 | 0.313 | < 0.001 | 0.200 |
| logC_max_ | 0.377 | 0.104 | 0.252 | 0.371 | < 0.001 | 0.027 |
| logC_min_ | 0.295 | 0.079 | 0.334 | 0.433 | < 0.001 | 0.056 |

Standard errors of the estimate range from 0.252 to 0.334 (without-rifampicin group). Thus, in 68% of patients (one standard error), the actual log-AUC_0-6h_, log-C_max_, and log-C_min_ will be within 0.253, 0.252, and 0.334 log units away from the value expected based solely on dose per kg. In other words, when attempting to base azithromycin dosing on the goal of achieving the average AUC0-6h of 1.83 (log=0.2625), patients will in actual fact have a 32% chance that their logAUC0-6h will be 0.253 log higher or lower: 0.00945 or 0.5155. This corresponds to a 32% chance that AUC0-6 is outside of the range 1.022 to 3.28

In the with-rifampicin group, the R-squared values are all lower and the standard errors of the estimates are higher, implying greater variability

# Figure S1 Scatterplots showing the relationship between log-dose per kg and log-AUC0-6h.


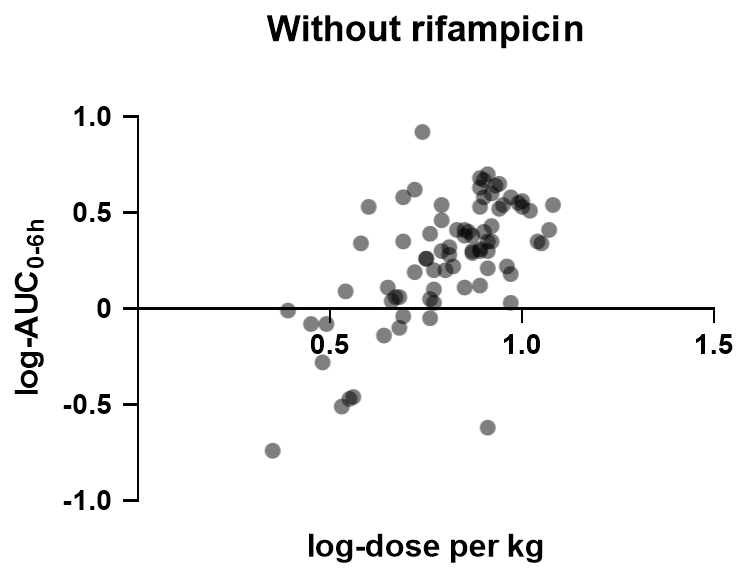

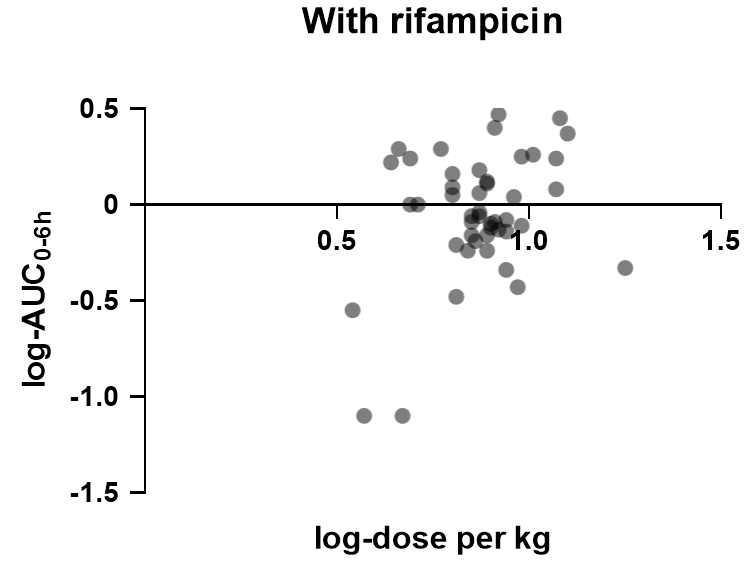


# Table S2. Multiple linear regression analysis

| **A** | Unstandardized coefficient | | Standardized coefficient | | Adjusted R-squared | p-value | |
| --- | --- | --- | --- | --- | --- | --- | --- |
|  | Rifampicin co-administration | LogDose/kg | Rifampicin use | LogDose/kg |  | LogDose/kg | Rifampicin co-administration |
| LogAUC_0-6h_ | -0.384 | 1.085 | -0.524 | 0.479 | 0.392 | <0.001 | <0.001 |
| logC_max_ | -0.393 | 1.122 | -0.506 | 0.463 | 0.364 | <0.001 | <0.001 |
| logC_min_ | -0.577 | 1.220 | -0.590 | 0.400 | 0.383 | <0.001 | <0.001 |

| **B** | Decrease (in %) due to rifampicin use* |
| --- | --- |
| AUC0-6h | 58.7 |
| Cmax | 59.5 |
| Cmin | 73.5 |

*Calculated using the unstandardized coefficient

**Supplementary Table 1**. Multiple linear regression for prediction of azithromycin exposure and the effect of rifampicin co-administration
